# Supplementary figures and images for: Beet Curly Top Iran Virus Rep and V2 Suppress Post-Transcriptional Gene Silencing via Distinct Modes of Action
Source: Viruses. 2023 Sep 26;15(10):1996. doi: 10.3390/v15101996 (PMC10611197; doi:10.3390/v15101996)

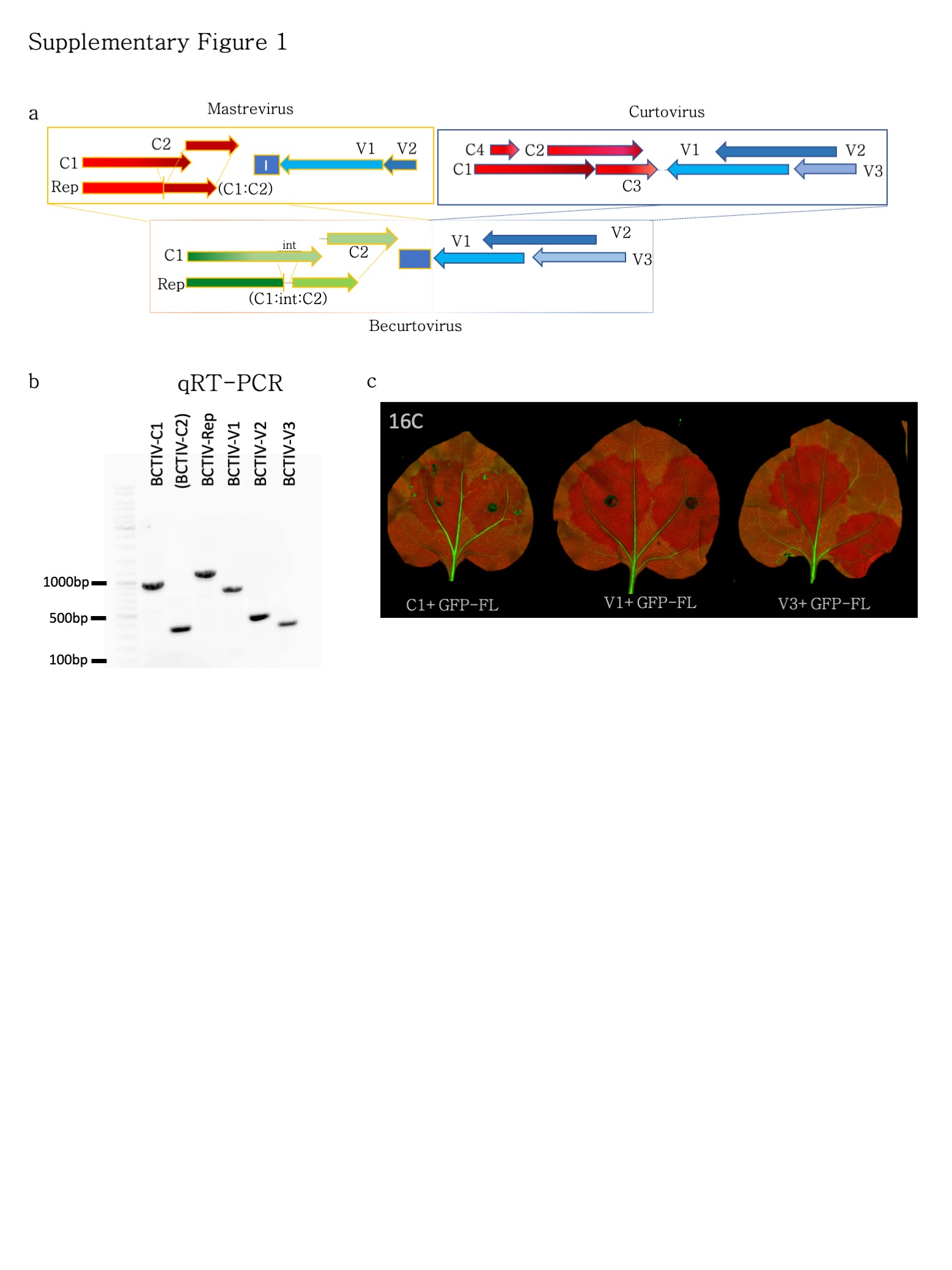

Supplement: Supplementary file 1 [file viruses-15-01996-s001.zip › Supplementary Figure 1.jpeg]

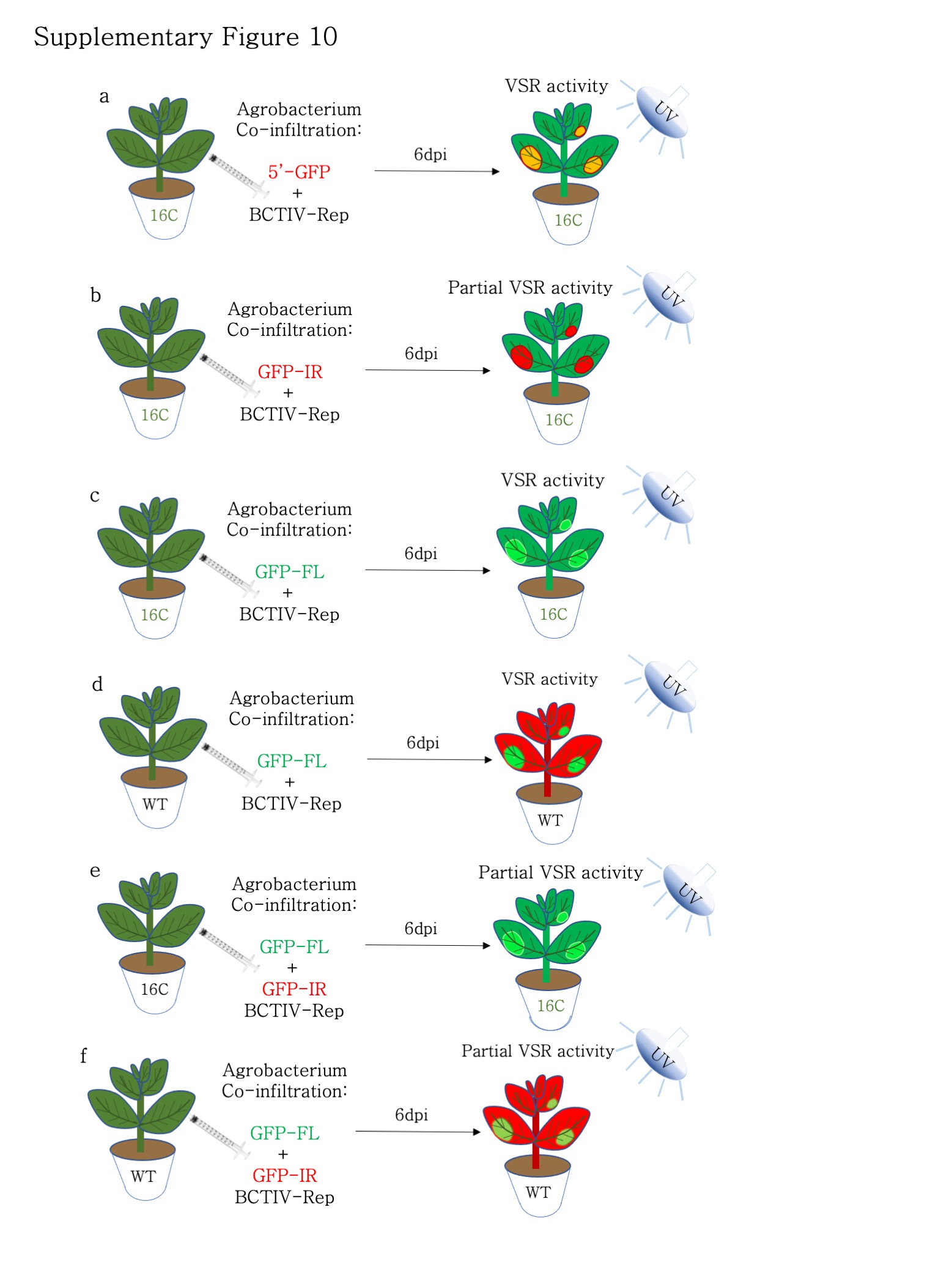

Supplement: Supplementary file 1 [file viruses-15-01996-s001.zip › Supplementary Figure 10.jpeg]

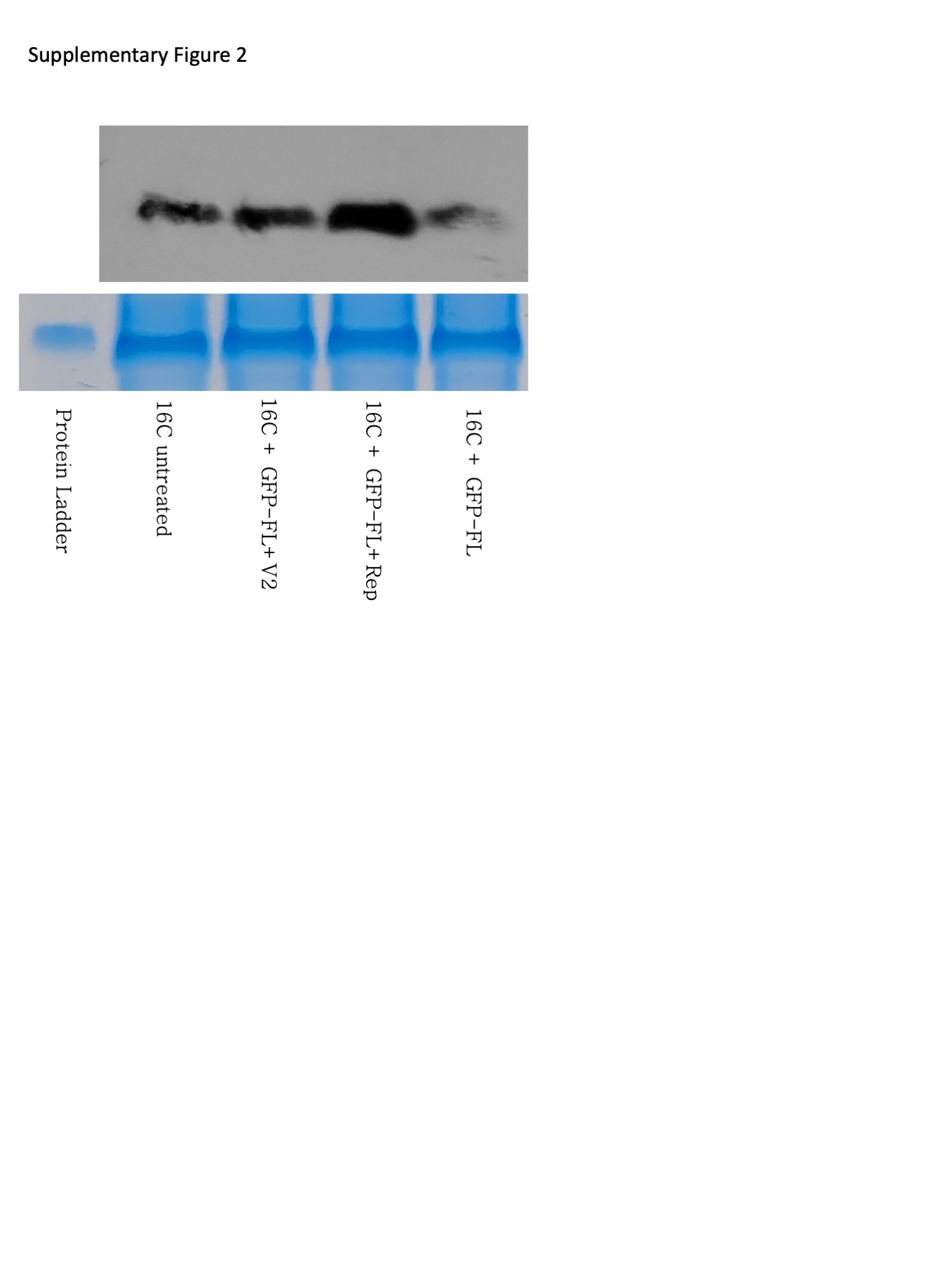

Supplement: Supplementary file 1 [file viruses-15-01996-s001.zip › Supplementary Figure 2.jpeg]

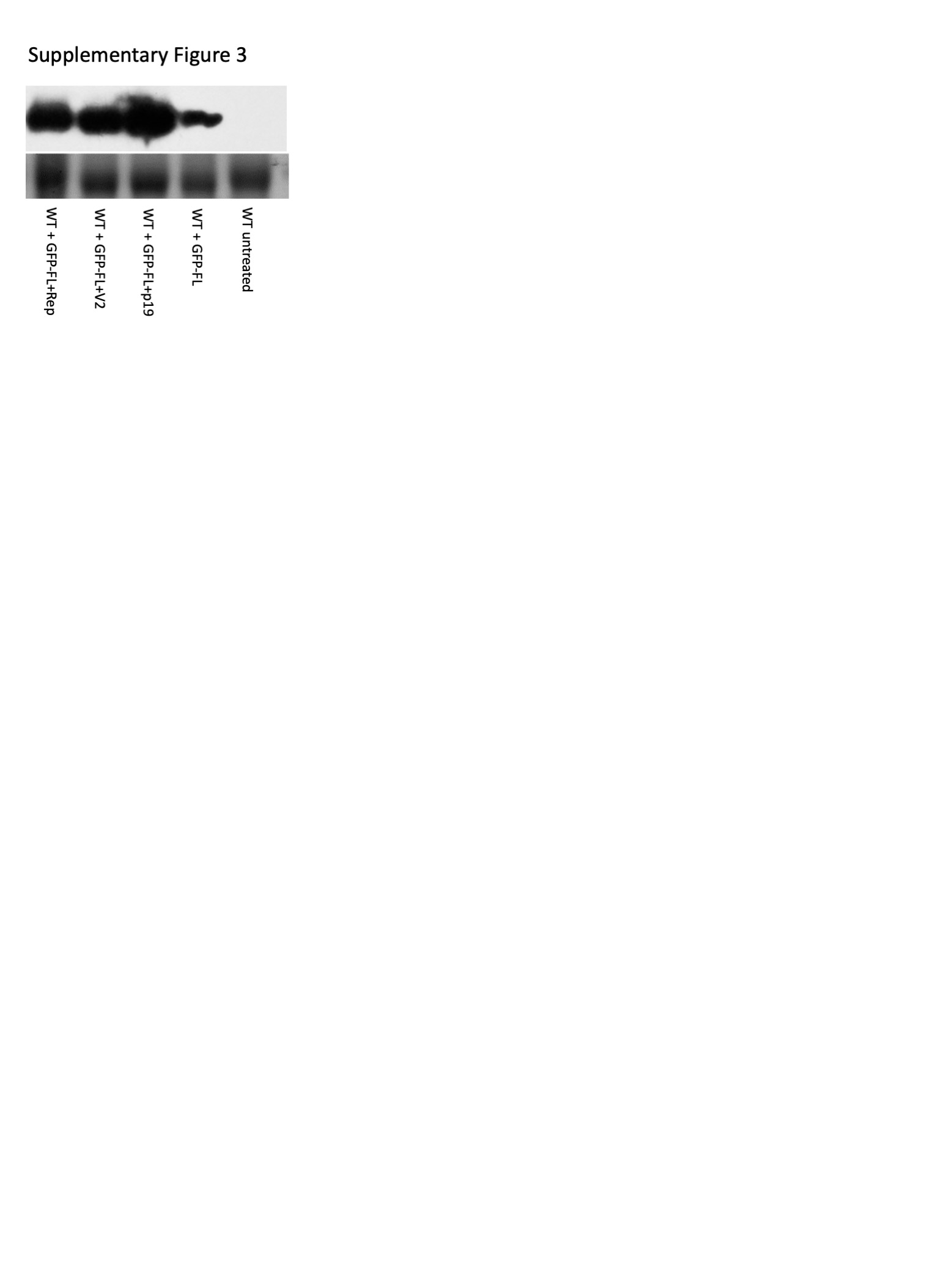

Supplement: Supplementary file 1 [file viruses-15-01996-s001.zip › Supplementary Figure 3.jpeg]

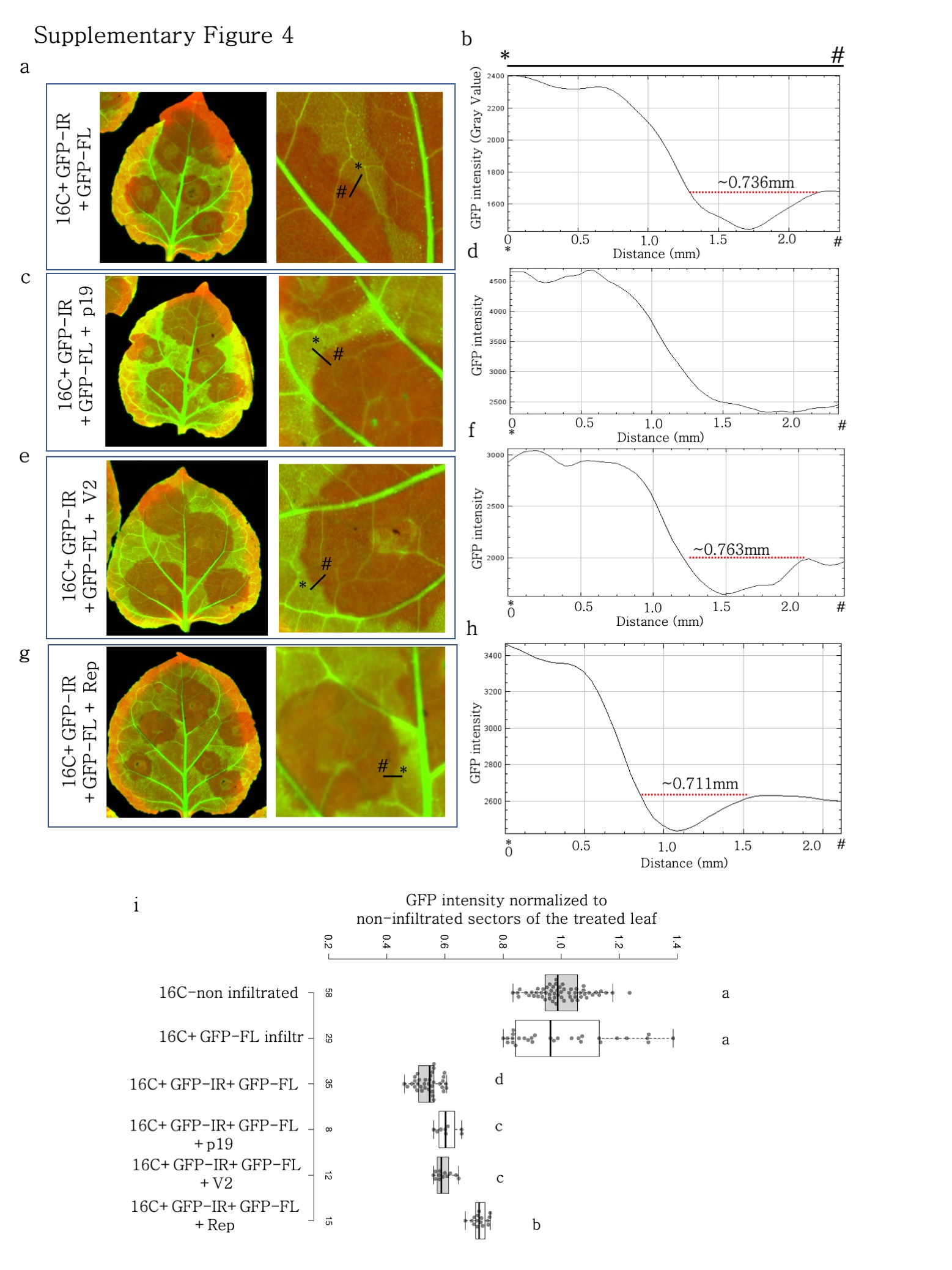

Supplement: Supplementary file 1 [file viruses-15-01996-s001.zip › Supplementary Figure 4.jpeg]

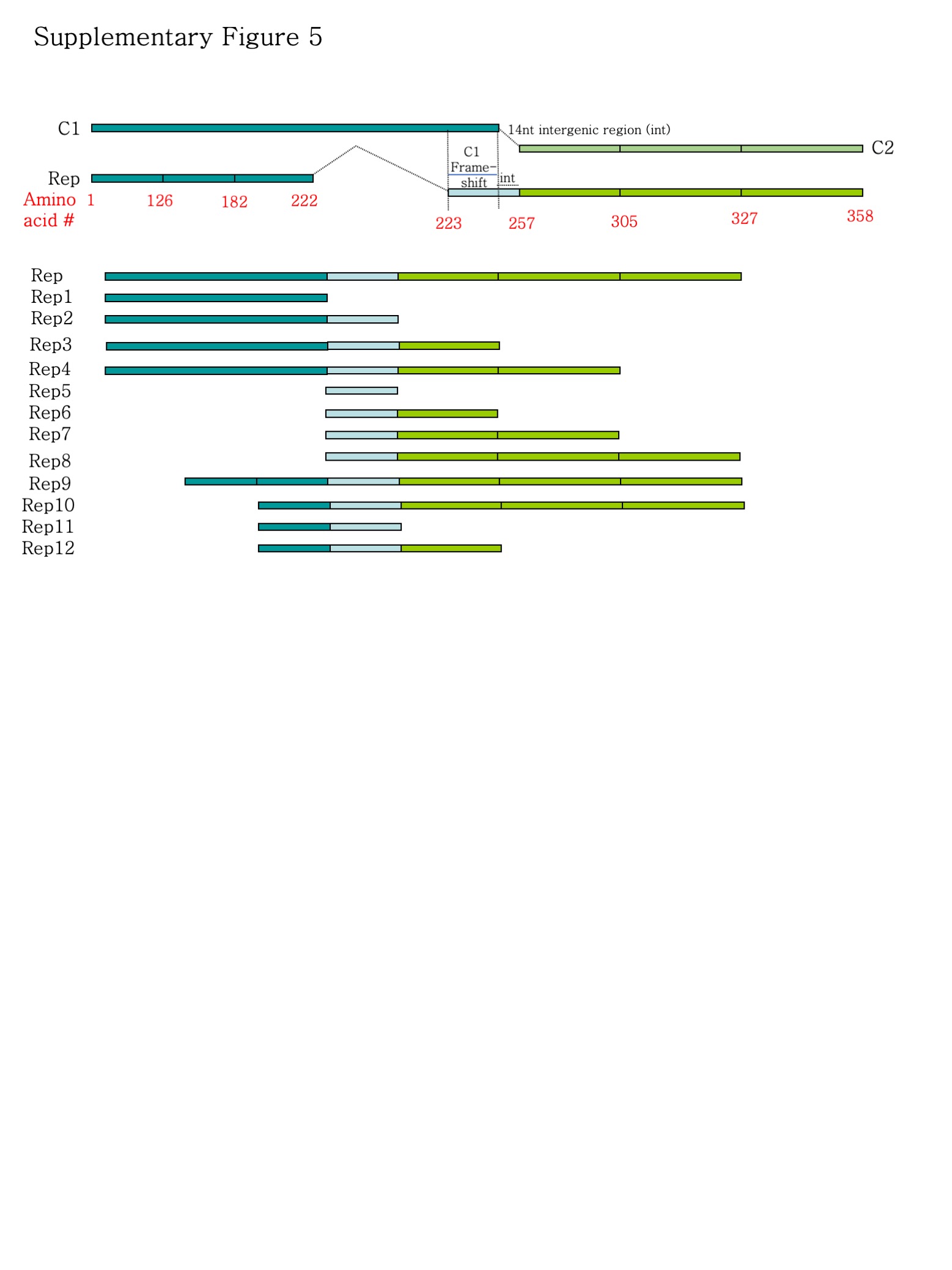

Supplement: Supplementary file 1 [file viruses-15-01996-s001.zip › Supplementary Figure 5.jpeg]

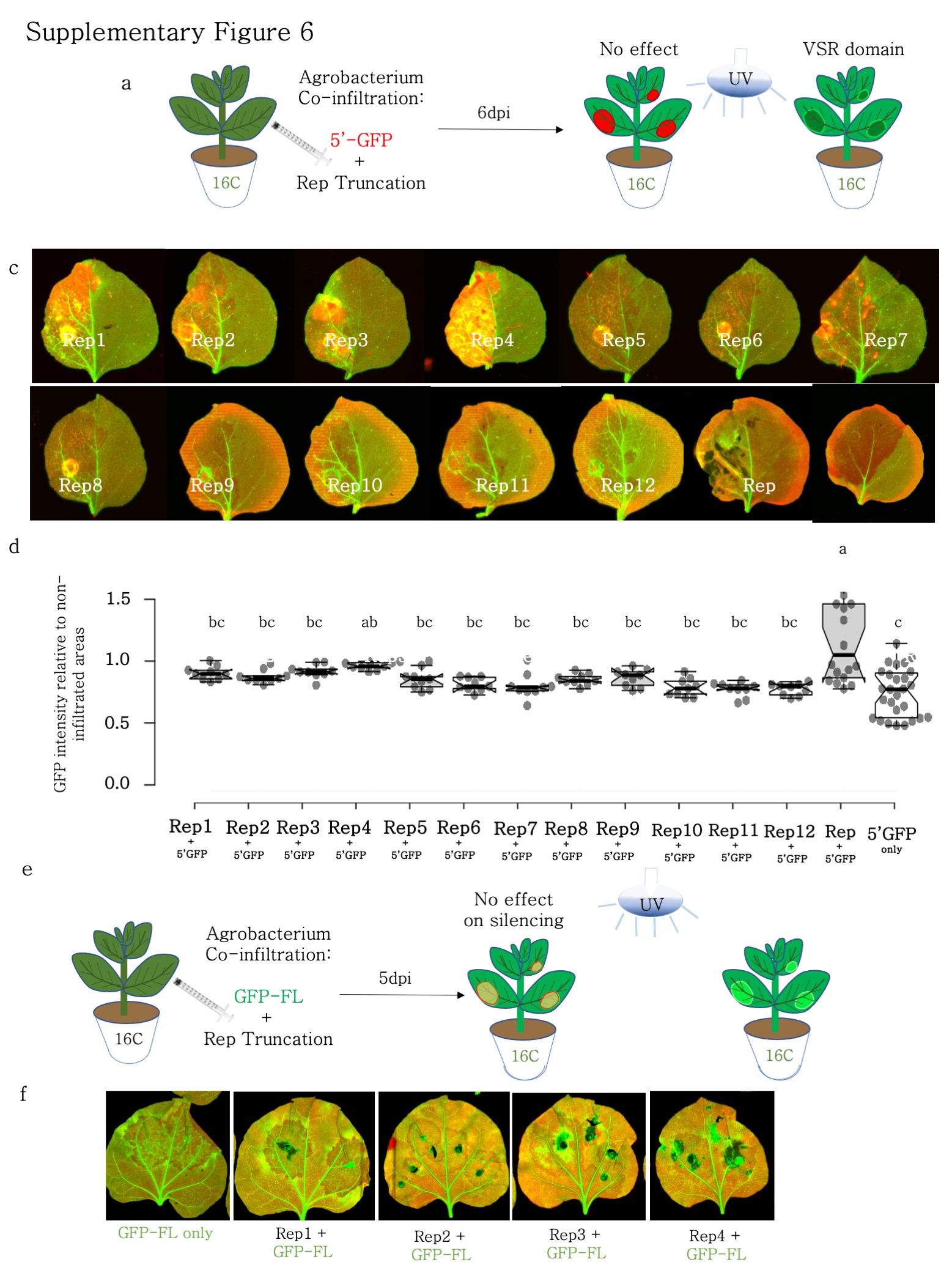

Supplement: Supplementary file 1 [file viruses-15-01996-s001.zip › Supplementary Figure 6.jpeg]

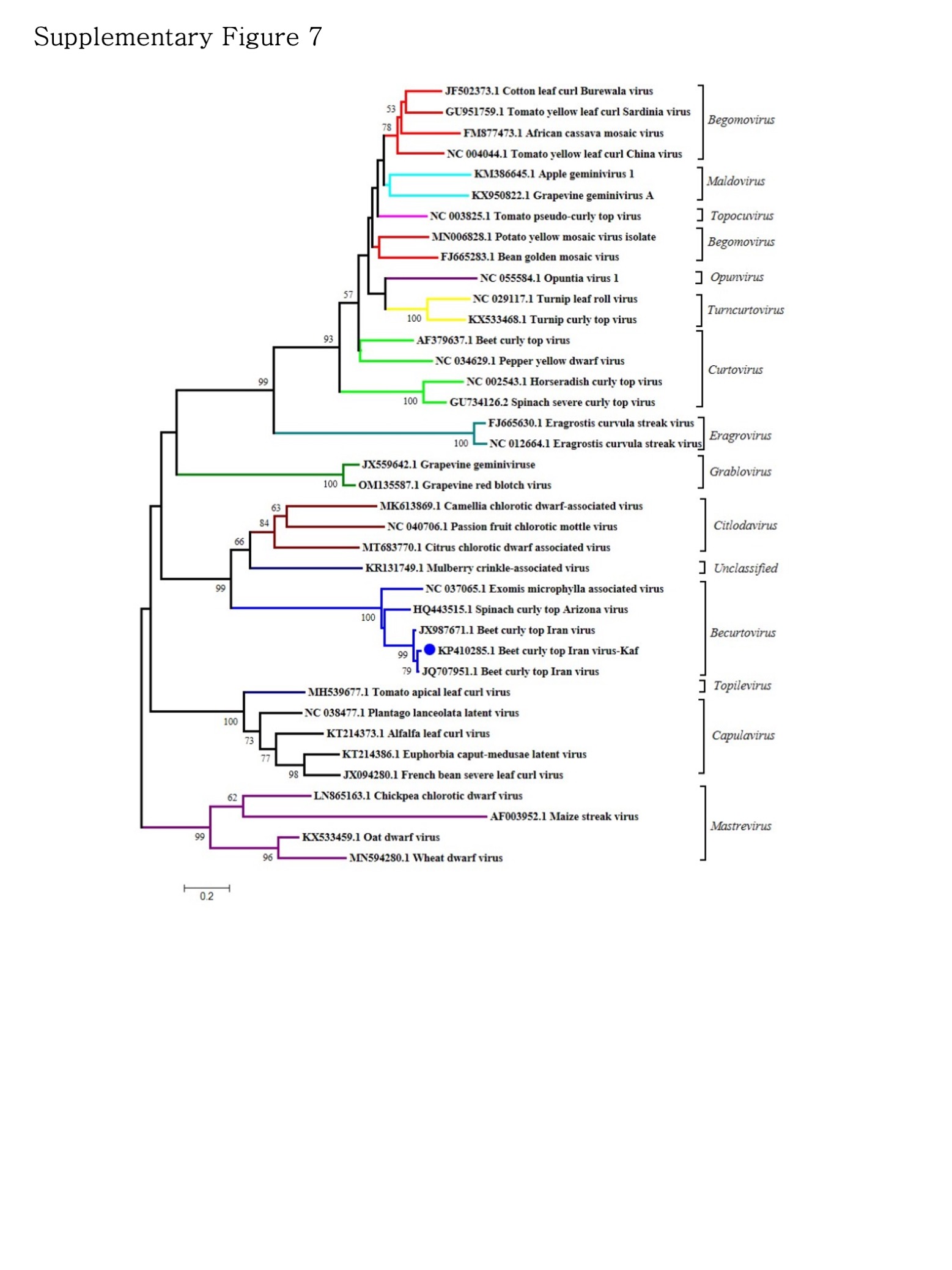

Supplement: Supplementary file 1 [file viruses-15-01996-s001.zip › Supplementary Figure 7.jpeg]

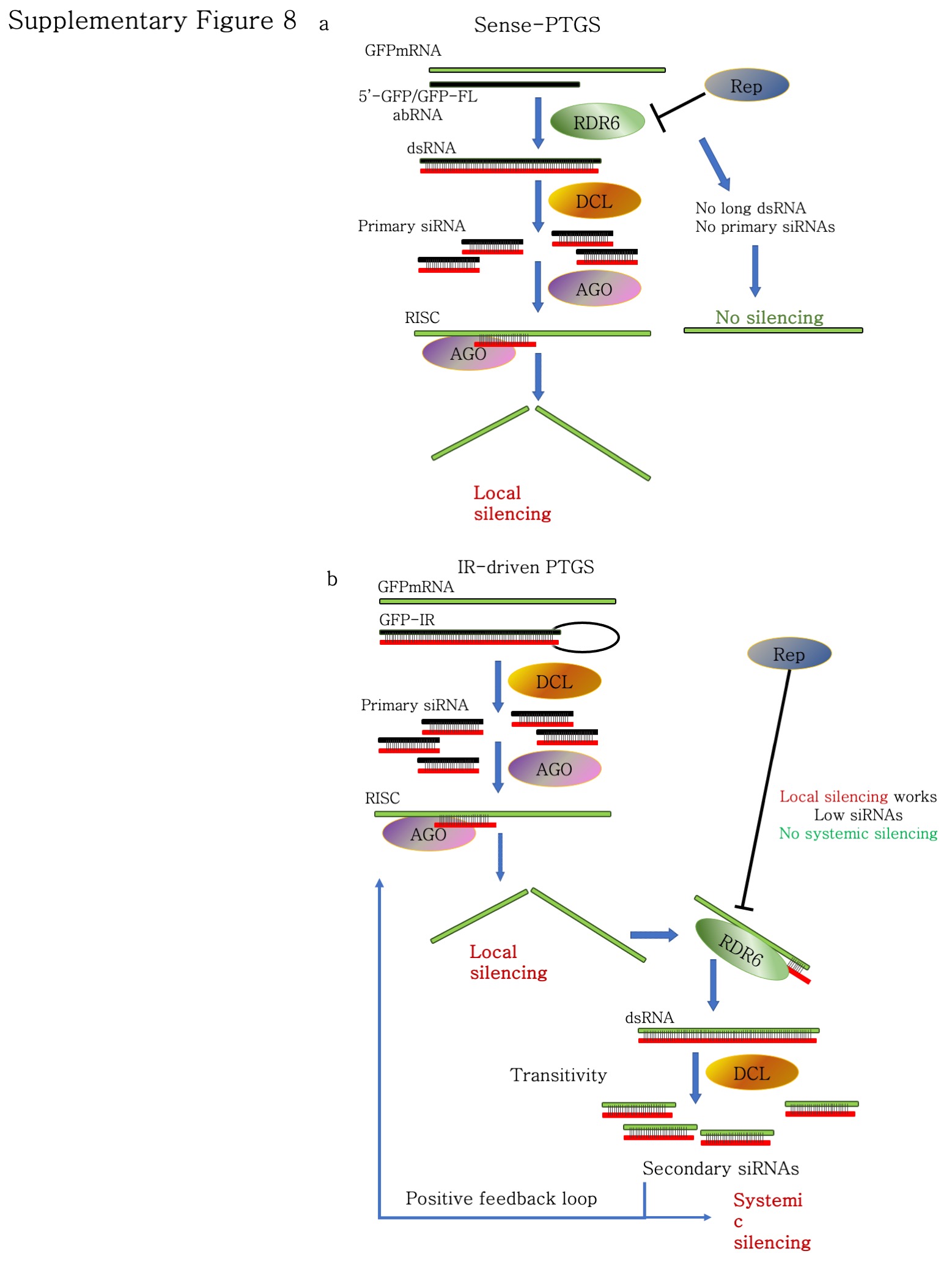

Supplement: Supplementary file 1 [file viruses-15-01996-s001.zip › Supplementary Figure 8.jpeg]

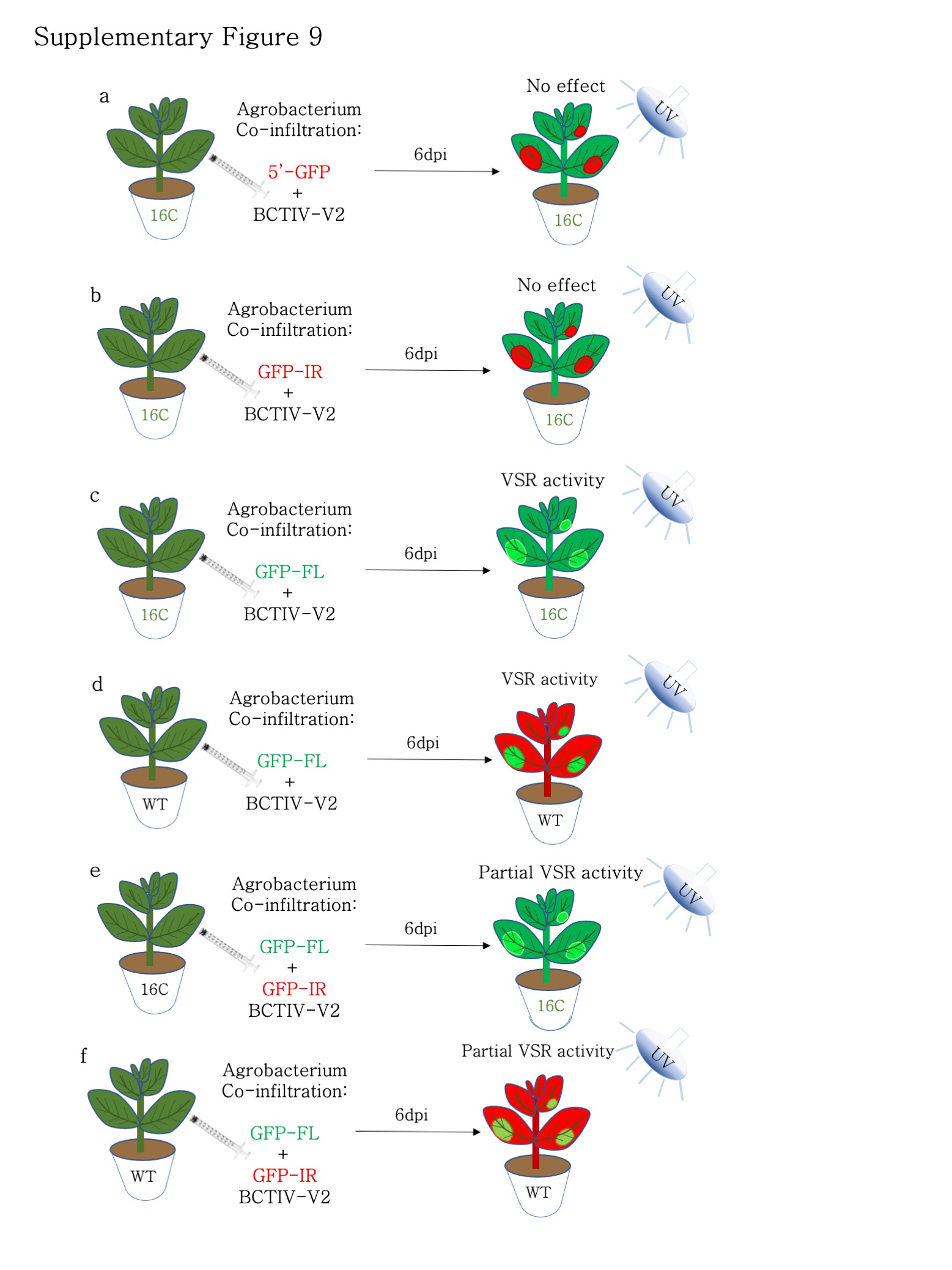

Supplement: Supplementary file 1 [file viruses-15-01996-s001.zip › Supplementary Figure 9.jpeg]
